# *Leuconostoc mesenteroides* mediates an electrogenic pathway to attenuate the accumulation of abdominal fat mass induced by high fat diet

**DOI:** 10.1038/s41598-020-78835-9

**Published:** 2020-12-14

**Authors:** Minh Tan Pham, John Jackson Yang, Arun Balasubramaniam, Adelia Riezka Rahim, Prakoso Adi, Thi Tra My Do, Deron Raymond Herr, Chun-Ming Huang

**Affiliations:** 1 Department of Biomedical Sciences and Engineering, National Central University, Taoyuan, 32001 Taiwan; 2 Department of Life Sciences, National Central University, Taoyuan, Taiwan; 3 Department of Pharmacology, National University of Singapore, Singapore, Singapore

**Keywords:** Microbiology, Bacteria

## Abstract

Although several electrogenic bacteria have been identified, the physiological effect of electricity generated by bacteria on host health remains elusive. We found that probiotic *Leuconostoc mesenteroides* (*L. mesenteroides*) can metabolize linoleic acid to yield electricity via an intracellular cyclophilin A-dependent pathway. Inhibition of cyclophilin A significantly abolished bacterial electricity and lowered the adhesion of *L. mesenteroides* to the human gut epithelial cell line. Butyrate from *L. mesenteroides* in the presence of linoleic acid were detectable and mediated free fatty acid receptor 2 (Ffar2) to reduce the lipid contents in differentiating 3T3-L1 adipocytes. Oral administration of *L. mesenteroides* plus linoleic acid remarkably reduced high-fat-diet (HFD)-induced formation of 4-hydroxy-2-nonenal (4-HNE), a reactive oxygen species (ROS) biomarker, and decreased abdominal fat mass in mice. The reduction of 4-HNE and abdominal fat mass was reversed when cyclophilin A inhibitor-pretreated bacteria were administered to mice. Our studies present a novel mechanism of reducing abdominal fat mass by electrogenic *L. mesenteroides* which may yield electrons to enhance colonization and sustain high amounts of butyrate to limit ROS during adipocyte differentiation.

## Introduction

Excessive abdominal fat accumulation leads to abdominal obesity (AO) which runs a higher risk of heart disease, hypertension, insulin resistance, and type 2 diabetes. The overall prevalence of AO was found to be 30–75% worldwide^1^. Strategies to improve access to prevention and treatment measures are urgently needed and would provide profound benefit in alleviating AO-associated diseases. Adipose tissue contributes to energy metabolism and lipid storage, and maintains 20% of the body’s glucose homeostasis^2^. Therefore, adopting a therapeutic approach with respect to new molecular targets and mechanisms in AO is of substantial interest.

Lipid accumulation caused by reactive oxygen species (ROS) production in adipocyte can be activated underlying pathophysiology^3,4^. ROS are both oxygen radicals and nonradicals that originate from mitochondrial respiration and enzymatic oxidases as by-products, which induce damage to proteins, lipids, and nucleic acids resulting in cellular dysfunction^5^. It has been reported that lipid accumulation is associated with systemic ROS in humans and animals, consequently leading to the development of age-related diseases^6^. Previous studies have revealed that the release of ROS was significantly higher in adipose tissue acquired from obese patients compared to non-obese healthy subjects^7^. Adipocyte-derived ROS contributes to cellular dysfunction that leads to the onset of metabolic disorders including obesity, insulin resistance, glucose uptake inhibition, and decreased expression of antioxidant enzymes, and upregulation of NADP(H) oxidases^8–11^.

Due to the likely role of ROS in mediating the pathologies of metabolic disease, the clinical use of high doses of oral or intravenous antioxidants has been explored. However, all of the antioxidant regimes used in patients with AO have had little effect on metabolic disease^12^, even in long-term antioxidant intervention trials^13^. Unexpectedly, harmful effects of antioxidants have been reported in literature, wherein some studies report a higher overall mortality rate in subjects treated with β-carotene or vitamin E^14–16^. Therefore, there is an increasing demand for the use of alternative modalities to control ROS in adipocytes. The beneficial activity of fermentation by gut bacteria has been extensively studied^17,18^. However, the effect of this process on scavenging lipid accumulation-induced ROS in adipocyte is still unclear.

Recently, several gut bacteria in the human microbiome, including Firmicutes phylum bacteria, have been found to be capable of electron production in vitro and in mice^19^*.* These electrogenic bacteria, also called metal-reducing bacteria, can oxidize organic compounds or hydrogen (H_2_) and provide electrons to minerals that contain ferric iron (Fe^3+^) or manganese (Mn^3+^ or Mn^4+^) as electron acceptors for respiration^20^. However, there have been no previous reports of the electrogenic capacity of *Leuconostoc mesenteroides* (*L. mesenteroides*). This fermentative Gram-positive bacterium belongs to the Firmicutes phylum and is a commensurate organism in the mammalian gut microbiome that is frequently associated with fermenting traditional functional foods^21,22^. Many *L. mesenteroides*-fermented products show antioxidant activity reducing the accumulation of ROS^18,22–24^. Despite the lack of reports of its electrogenic potential, *L. mesenteroides* possessed ferric iron reductase activity^23^. Moreover, it was documented that *L. mesenteroides* expresses intracellular cyclophilin A with activity of peptidyl-prolyl cis–trans isomerase, acting as an electron donor for antioxidant enzymes^25,26^. The function of cyclophilin A as a chaperone protein that is involved in protein folding and the maintenance of various cellular processes suggests its essential role in bacterial resistance to ROS stress^27^.

It has been reported that short-chain fatty acids (SCFAs) including butyrate in fermentation metabolites of gut bacteria exert multiple positive effects on human health. For instance, buyrtate can contribute to improving insulin sensitivity^28^ and controlling obesity^29^. In this study, we found that *L. mesenteroides* can produce electricity and ample butyrate in the process of metabolizing linoleic acid (C18:2), a long-chain polyunsaturated fatty acid^30^. Electricity production may enhance the bacterial colonization, leading to high amounts of SCFAs that can be sustained in the gastrointestinal tract and bloodstream. We further showed that cyclophilin A is an essential protein for electricity production in *L. mesenteroides.* Our study revealed a cyclophilin A-mediated electricity production pathway of butyrate-producing *L. mesenteroides* for protection against pathological adipogenesis and its associated AO.

## Materials and methods

### Ethics statement

All animal experimental protocols were approved by National Central University (NCU), Taiwan. The methods were carried out in accordance with the Institutional Animal Care and Use Committee (IACUC) of the National Central University (NCU), Taiwan (NCU-106-016, 19 December 2017). Institute Cancer Research (ICR) female mice (8–9 weeks old) were obtained from the National Laboratory Animal Center, Taipei, Taiwan for all experiments. Mice were sacrificed via inhalation of CO_2_ anesthesia.

### Bacterial culture

*Leuconostoc mesenteroides* EH-1 strain was originally isolated from Mongolian curd cheese. The bacteria were cultured in tryptic soy broth (TSB) (Sigma, St. Louis, MO, USA) overnight at 37 °C. The cultures were diluted 1:100 and cultured to an optical density 600 nm (OD_600_) = 1.0. Bacteria were harvested by centrifugation at 5000 rpm for 10 min, washed with phosphate-buffered saline (PBS), and suspended in PBS for further experiments.

### Electricity detection

A lab-fabricated chamber equipped with a cathode and anode was used for the in vitro detection of bacterial electricity. A carbon filth (2.5 cm × 10 cm) (Homy Tech, Taoyuan, Taiwan) served as an anode. A carbon cloth (10 cm × 10 cm) (Homy Tech) covered with a nafion (sulfonated tetrafluoroethylene based fluoropolymer-copolymer) membrane N117 (6 cm × 6 cm) (Homy Tech), a proton exchange membrane (PEM), functioned as a cathode. Anode and cathode were connected by copper wires, which sequentially were attached to 1000 Ω external resistance. *L. mesenteroides* EH-1 bacteria [10^7^ colony-forming unit (CFU)/ml] in rich media [10 g/l yeast extract (Biokar Diagnostics, Beauvais, France), 5 g/l TSB, 2.5 g/l K_2_HPO_4_ and 1.5 g/l KH_2_PO_4_] in the absence of presence of 2% linoleic acid were pipetted on the surface of the anode. *L. mesenteroides* EH-1 in rich media was pretreated with 2 µM TMN355 (Santa Cruz Biotechnology, Dallas, TX, USA), an inhibitor of cyclophilin A dissolved in 2% final concentration of dimethyl sulfoxide (DMSO), at 37 °C for 24 h followed by washing twice with 1 × PBS. The changes in voltage (mV) against time (min) were recorded by a digital multimeter (Lutron, DM-9962SD, Sydney, Australia). The voltage was recorded every 10 s to plot a graph of voltage against time.

### Real-time polymerase chain reaction (PCR)

A StepOnePlus Real-time PCR System (ThermoFisher Scientific, Waltham, MA, USA) using Power SYBR Green and PCR Master Mix (ThermoFisher Scientific) was used to examine gene expression of the cyclophilin A in *L. mesenteroides* EH-1 treated with/without 2 μM TMN355 (Bio-Techne Corporation, Minneapolis, MN, USA). RNA (1 ng) was converted into cDNA using an iScript cDNA Synthesis kit (Bio-Rad, Hercules, CA, USA). cDNA (50 ng/μl) of *L. mesenteroides* EH-1 was used as a template. Primers were designed using the Primer-Blast tool from the National Center for Biotechnology Information (NCBI). The reaction conditions were set for 40 cycles as follows: 95 °C for 10 min followed by 95 °C for 15 s, 48 °C for 60 s, and 72 °C for 30 s. A complete reaction was achieved with three biological replicates, and each sample consisted of three technical replicates. The gene expression of triosephosphate isomerase (tpi) was used for normalization. The relative expression levels were analyzed using the cycle threshold (2^−ΔΔCt^) method. Primers included forward 5′ TCCAAACTAGGATAGCCGCC 3′ and reverse 5′ TTCGTGGCGCTGTTTCAATG 3′ for cyclophilin A; and forward 5′ ACCCTCAGTGGCTCAAGTGG 3′ and reverse 5′ GGCCAGCGTCTGACGTATCA 3′ for tpi.

### Ferrozine assays

*Leuconostoc mesenteroides* EH-1 (10^7^ CFU/ml) was pretreated with or without 2 μM TMN355. After centrifugation, bacterial pellets were resuspended in rich media supplemented with 2% linoleic acid and 0.5 mmol/l flavin mononucleotide (FMN, Sigma) before addition of 4 mmol/l ferrozine (Alfa Aesar Chemicals, Tewsbury, MA, USA). Experiments were initiated by adding 100 μl of bacteria to an equivalent volume of 50 mmol/l ferric ammonium citrate (Sigma) and conducted in triplicate at 37 °C in a 96-well format using a plate reader. OD_562_ measurements were made after 37 °C incubation for 30 min. The color change of media containing ferrozine and ferric ammonium citrate was quantified by a calibration curve.

### Gas chromatography mass spectrometry (GC–MS) analysis

*Leuconostoc mesenteroides* EH-1 (10^7^ CFU/ml) was incubated in 10 ml rich media in the presence of 2% linoleic acid for 24 h. After centrifugation at 5000 × *g* for 10 min, bacteria in supernatants were further removed by 0.22 µm filters. SCFAs in the fermentation media were detected by ethyl acetate (Residue Analysis OmniSolv, EMD Millipore, Billerica, MA) liquid–liquid extraction after addition of 50 µl of ^2^H_7_-butyrate (1 mg/ml) (C/D/N Isotopes, Quebec, Canada) as an internal standard, acidification with 0.5% ortho-phosphoric acid (ThermoFisher Scientific) and saturation with sodium chloride (ThermoFisher Scientific) followed by GC–MS analysis using an Agilent 5890 Series II GC in conjunction with 5971 MS detector (Agilent Technologies, Inc., Palo Alto, CA). A 70 eV electron was utilized for ionization. The levels of SCFAs in the fermentation media were quantified by a calibration curve made from six non-zero levels using the Free Fatty Acids Test Standard (Restek Corporation, Bellefonte, PA) which was diluted 500-, 1000-, 2000-, 5000-, and 10,000-folds.

### Bacterial adhesion assay

*Leuconostoc mesenteroides* EH-1 bacteria were centrifuged at 14,000 rpm at 4 °C for 5 min, washed four times with 1 × sterilized PBS. After washing, bacteria were resuspended at a concentration of 10^7^ CFU/ml bacteria in Hepes-supplemented Dulbecco's Modified Eagle Medium (DMEM). Confluent Caco-2 cells, the human epithelial colorectal adenocarcinoma cells, in a 96 microplate were incubated with *L. mesenteroides* EH-1 (100 µl/well) at 37 °C, 5% CO_2_ for 1 h. The non-adhered bacteria were removed by washing the cells twice with 2 ml Hepes-supplemented DMEM. The Caco-2 cells were further incubated with 100 µl trypsin-ethylenediaminetetraacetic acid (EDTA) at 37 °C for 15 min. After that, 100 µl DMEM supplemented with 10% fetal bovine serum (FBS) (Irvine Scientific, Santa Ana, CA, USA) was added to each well to stop trypsin–EDTA reaction. Caco-2 cells with adhered *L. mesenteroides* EH-1 were detached by repeated pipetting. Serial fivefold dilutions of cells and bacteria were spotted on a TSB agar plate for 48 h. The colonies on the plate were count to determine the bacterial CFUs.

### Cell culture and differentiation

3T3-L1 preadipocytes (ATCC CL-173) were cultured in culture media with DMEM (ThermoFisher Scientific), 1% penicillin–streptomycin (10,000 µ/ml) (ThermoFisher Scientific), and 10% FBS, 10 mmol/l l-glutamine (Sigma), 1 mmol/l sodium pyruvate (Sigma). Differentiation was induced by treatment of post-confluent cells with differentiation medium A (DMA) consisting of DMEM, 0.5 mmol/l 1-methyl-3-isobutylxanthine (IBMX), 1.0 µM dexamethasone (DEX), 1 µM insulin (Sigma), 1% penicillin–streptomycin, and 10% FBS. Four days after the initiation of differentiation, the DMA was replaced with the differentiation medium B (DMB) consisting of DMEM, 1.0 µM insulin, 1% penicillin–streptomycin, and 10% FBS. Each medium was then refreshed every 2 days on Days 0, 2, 4, and 6. Supernatant (100 µl/ml) of the *L. mesenteroides* EH-1 bacteria in TSB media with or without 2% linoleic acid was added onto cell culture at 37 °C for 30 min, then replaced with differentiation media on Days 1, 3, 5, and 7. The supernatant from media containing Supernatants were obtained by filtering the bacterial media using a Whatman nylon membrane with a 0.45 µm pore size (GE Healthcare, Chicago, IL, USA). In some experiments, 3T3-L1 preadipocytes were treated with 100 µM butyrate in the presence of 0.1 µM GLPG-0974 (Tocris Bioscience, Bristol, UK) in 0.1% dimethylsulfoxide (DMSO). 3T3-L1 preadipocytes were treated with butyrate in the presence of 0.1% DMSO served as a control.

### Oil Red O staining

The amount of lipid accumulation in 3T3-L1 preadipocytes was detected by an Oil Red O staining kit (Sigma). In brief, cells fixed in 4% formaldehyde were stained with an Oil Red O working solution for 30 min. Lipids stained red were imaged by light microscopy and extracted in 250 µl isopropanol for quantification via measuring absorbance at 510 using a Synergy HTX plate reader (BioTek Instruments, Winooski, VT, USA)^31^.

### Measurement of intracellular ROS

Production of ROS in 3T3-L1 preadipocytes was determined using 2,7-diacetyl dichlorofluorescein (DCFH-DH) (ThermoFisher Scientific) that can penetrate into the intracellular matrix of cells, where it was oxidized by ROS to fluorescent dichlorofluorescein (DCF)^32^. Cells were washed two times with DMEM. 1 ml aliquot of cells mixed with 1 μl DCFH-DA (1 mg/ml) was incubated at 37 °C for 30 min under dark condition. Fluorescence was measured with excitation and emission at 485 and 530 nm, respectively using a multimode reader (Infinite 200 Pro, Tecan Group Ltd., Männedorf, Switzerland). The ROS-emitted viable cells were observed under fluorescence microscope (Micro-shot Technology Limited, Guangzhou, China).

### High-fat-diet (HFD) fed mice

Mice were fed with a standard vivarium-provided chow diet (SCD) (BioLASCO Taiwan Co., Ltd., Taipei, Taiwan; 5% fat, 24% protein, and 54% carbohydrate) or 60% calorie HFD (60% fat by wt., BioLASCO Taiwan Co., Ltd.) and administered 200 µl of 2% linoleic acid, *L. mesenteroides* EH-1 (10^7^ CFU) or *L. mesenteroides* EH-1 plus linoleic acid by oral gavage at an interval of 3 days for 6 weeks. Some mice were fed with HFD and administered TMN355 (2 µM, Sigma)-pretreated *L. mesenteroides* EH-1 plus linoleic acid. Body weights were measured weekly. The abdominal fat mass was photographed and homogenized for analysis of 4-hydroxy-2-nonenal (4-HNE) by western blotting. Five mice per group were used in each experiment.

### Western blotting

The abdominal fat mass (100 mg) of ICR mice was homogenized in 400 µl lysis buffer containing 4 µl protease inhibitor and 4 µl 0.5 M EDTA (ThermoFisher Scientific). Homogenates were subjected to 10% sodium dodecyl sulfate polyAcrylamide gel electrophoresis (SDS‐PAGE), transferred to a polyvinylidene difluoride (PVDF) membrane (Sigma) and blocked with 5% (*w/v*) non‐fat milk before incubation overnight with primary antibodies to 4-HNE (1:2,000, Abcam, Cambridge, MA, USA) or β‐actin (1:1,000, Cell Signaling, Danvers, MA, USA). This was followed by treatment for 1 h with horseradish peroxidase (HRP)‐conjugated secondary antibody (goat anti‐mouse (1:5000). Protein bands were detected with a chemiluminescent detection reagent and Omega Lum C Imaging System (Gel Company, San Francisco, CA, USA). Densitometric analysis of the protein bands was performed using Image J software (National Institute of Health (NIH), Bethesda, MD, USA).

### Statistical analysis

Data analysis was conducted by unpaired t-test using GraphPad Prism 5 (GraphPad Software, Inc., San Diego, USA). The *p*-values of < 0.05 (*), < 0.01 (**), and < 0.001 (***) were considered significant. The mean ± standard deviation (SD) for at least three independent experiments was calculated.

## Results

### Electricity and SCFAs were produced by *L. mesenteroides* EH-1 plus linoleic acid

**Figure 1:**
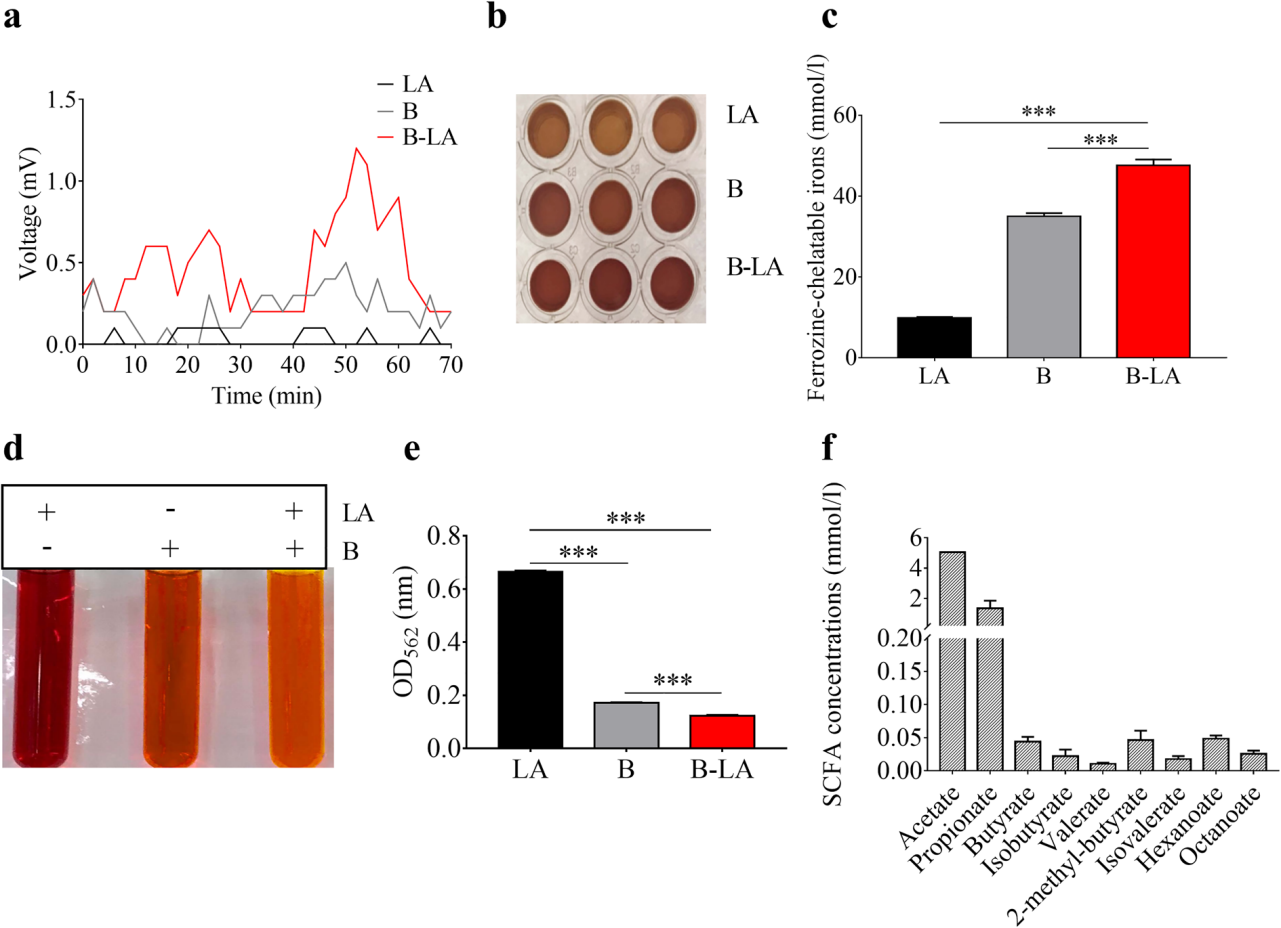
Characterization of electricity produced by *L. mesenteroides* EH-1 bacteria in the presence of linoleic acid. (**a)** Chronoamperometry results were obtained from an electrochemical chamber with *L. mesenteroides* EH-1 bacteria alone (B), 2% linoleic acid (LA), bacteria plus linoleic acid (B-LA). The changes in voltage (mV) against time (min) were recorded by Lutron software. **(b)** Ferrozine and ferric ammonium citrate were added into media with linoleic acid (LA), *L. mesenteroides* EH-1 bacteria (B), bacteria plus linoleic acid (B-LA). The dark brown complexes of ferrozine-chelatable irons (mmol/l) were photographed. (**c**) The OD_562_ values of ferrozine-chelatable irons (mmol/l) were quantified. (**d**) Linoleic acid fermentation of *L. mesenteroides* EH-1. *L. mesenteroides* EH-1 (B) was incubated in rich media with/without linoleic acid (LA) for 24 h. Rich media plus linoleic acid (LA) without *L. mesenteroides* EH-1 were included as controls. (**e**) Fermentation was detected by OD_562_. (**f**) The concentrations of nine SCFAs were detected by GC–MS analysis. Data shown represent the mean ± SD of experiments performed in triplicate. ***p < 0.001 (two-tailed *t*-test by GraphPad Prism 5).

In the presence of a variety carbon sources, several probiotic bacteria are able to yield acetate and butyrate which are known to be electron donors in a microbial fuel cell system^33–35^. We thus examined the eletrogenicity of probiotic *L. mesenteroides* EH-1 strain in the presence of 2% linoleic acid as a carbon source. An in vitro chamber with cathode and anode electrodes was fabricated to detect bacterially generated electricity. As shown in Fig. 1a, little or no voltage change was recorded over a monitoring period of 70 min in media with linoleic acid alone. A slight increase in voltage was detected in the media with *L. mesenteroides* EH-1 alone. The voltage was considerably raised to a peak of more than 1 mV when bacteria were placed in media in the presence of 2% linoleic acid. These data demonstrate that *L. mesenteroides* EH-1 is an electrogenic bacterium. We next examined the effect of bacterial electricity on regulation of the redox cycling of iron. In a ferrozine assay, linoleic acid, *L. mesenteroides* EH-1 or *L. mesenteroides* EH-1 plus linoleic acid were added into a reaction solution containing FMN, ferrozine and ferric (Fe^3+^) ammonium citrate. As shown in Fig. 1b,c, the concentration of ferrozine-chelatable iron (dark brown complex) in the reaction solution containing *L. mesenteroides* EH-1 plus linoleic acid was markedly higher than in the reaction solution containing linoleic acid alone or bacteria alone. This result suggests that electrons produced by *L. mesenteroides* EH-1 plus linoleic acid converted Fe^3+^ to ferrozine-chelatable iron. To determine whether SCFAs were produced in the culture of *L. mesenteroides* EH-1 plus linoleic acid, *L. mesenteroides* EH-1 was cultured in rich media in the presence of 2% linoleic acid for 24 h. Rich media with linoleic acid alone or *L. mesenteroides* EH-1 alone served as controls. The media in the culture of *L. mesenteroides* EH-1 with linoleic acid turned yellow after incubation for 24 h (Fig. 1d). As shown in Fig. 1c, the OD_562_ of media with *L. mesenteroides* EH-1 plus linoleic acid demonstrated significant decreases compared to controls, indicating that linoleic acid was fermented by *L. mesenteroides* EH-1. GC–MS analysis was performed to quantify the level of SCFAs in fermentation media of *L. mesenteroides* EH-1. Nine SCFAs including acetate, propionate, and butyrate were detectable in media from linoleic acid fermentation of *L. mesenteroides* EH-1 (Fig. 1f).

### Adipocyte differentiation was attenuated by fermentation media of *L. mesenteroides* and butyrate

**Figure 2:**
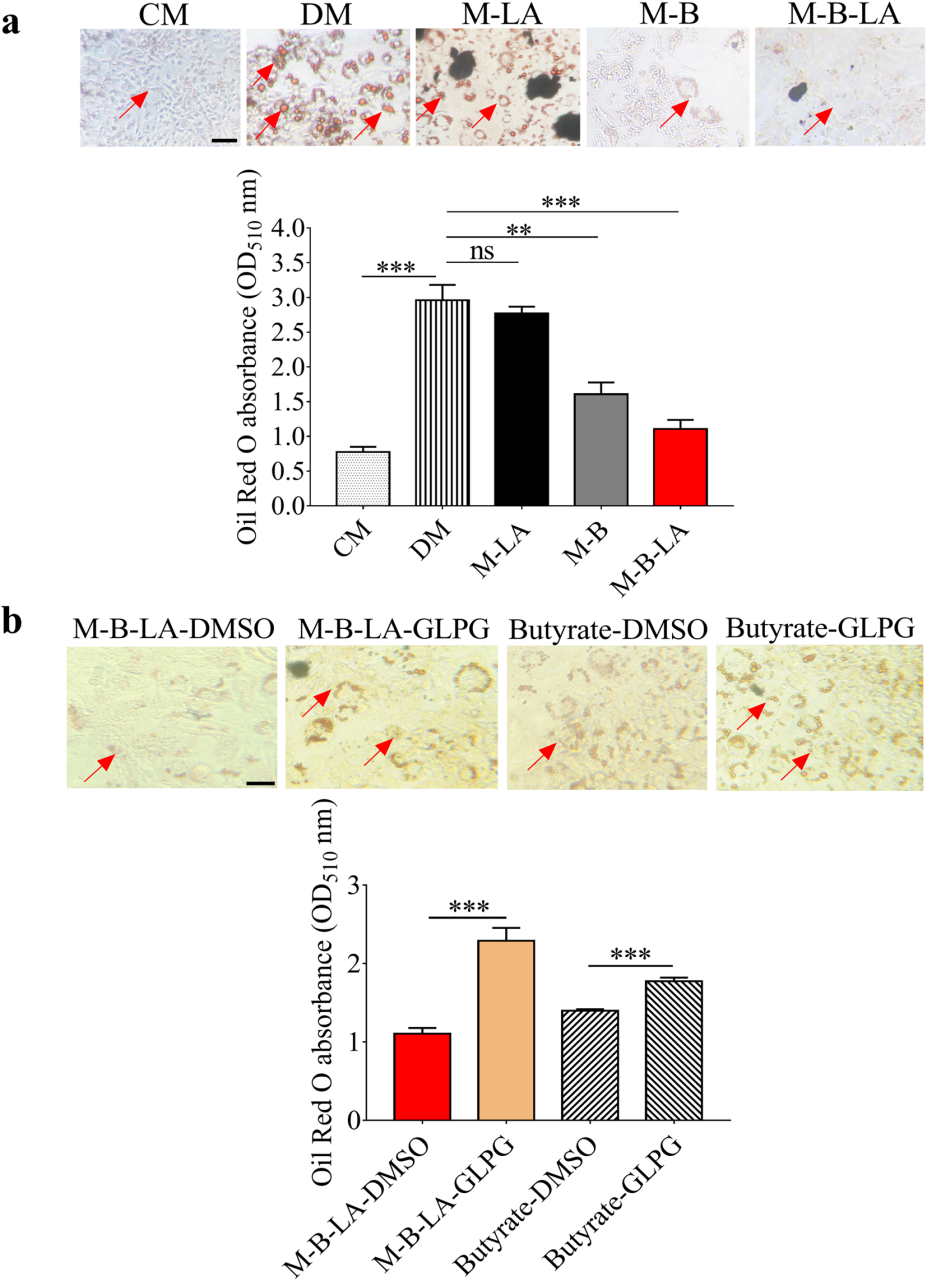
Effect of linoleic acid fermentation of *L. mesenteroides* EH-1 and butyrate on adipocyte differentiation. (**a**) From left to right: 3T3-L1 preadipocytes were treated with culture media (CM), differentiation media (DM), differentiation media with rich media containing linoleic acid (M-LA) or supernatant from the culture of *L. mesenteroides* EH-1 bacteria alone (M-B), bacteria plus linoleic acid (M-B-LA). Lipids (arrows) were stained with Oil Red O and extracted by isopropanol for quantification by absorbance at OD_510_. (**b**) From left to right: 3T3-L1 preadipocytes were treated with supernatant from the culture of bacteria plus linoleic acid in the presence of GLPG-0974 (GLPG), a Ffar2 antagonist (M-B-LA-GLPG), or DMSO (M-B-LA-DMSO). Preadipocytes in differentiation media treated with butyrate (Butyrate-DMSO) or a Ffar2 antagonist (Butyrate-GLPG) were included. Data are the mean ± SD of experiments performed in triplicate. **p < 0.001. ***p < 0.0001. *ns* non-significant (two-tailed *t*-test by GraphPad Prism 5). Bars = 100 µm.

To explore if linoleic acid fermentation of *L. mesenteroides* EH-1 affects adipocyte differentiation, we added the supernatant from the culture of *L. mesenteroides* EH-1 plus linoleic acid onto the differentiating 3T3-L1 preadipocytes. Lipid accumulation during 3T3-L1 differentiation were detected by Oil Red O staining. The content of lipids was significantly increased during cell differentiation when the culture media of 3T3-L1 preadipocytes were replaced with differentiation media (Fig. 2a). The differentiation-induced increase in lipids were markedly inhibited by addition of supernatant of the culture of *L. mesenteroides* EH-1 plus linoleic acid. There was no change in lipid content in differentiated 3T3-L1 cells after addition of media containing linoleic acid alone, although inhibition of lipid production was observed by adding the supernatant of the culture of *L. mesenteroides* EH-1 alone. To examine the contribution of SCFAs to adipocyte differentiation, the differentiated 3T3-L1 cells treated with supernatant of the culture of bacteria plus linoleic acid were added with GLPG-0974, a free fatty acid receptor 2 (Ffar2) antagonist. Inhibition of Ffar2 by GLPG-0974, not its DMSO solvent, significantly diminished the action of supernatant of the culture of bacteria plus linoleic acid on the inhibition of lipid production (Fig. 2b). Furthermore, cells treated with butyrate markedly reduced lipid contents. The reduction can be reversed by addition of GLPG-0974. Results above clearly demonstrated that Ffar2 mediated the effect of butyrate, one of SCFAs produced by linoleic acid fermentation of *L. mesenteroides* EH-1 alone, on adipocyte differentiation.

### The formation of ROS and 4-HNE was suppressed by *L. mesenteroides* EH-1

**Figure 3:**
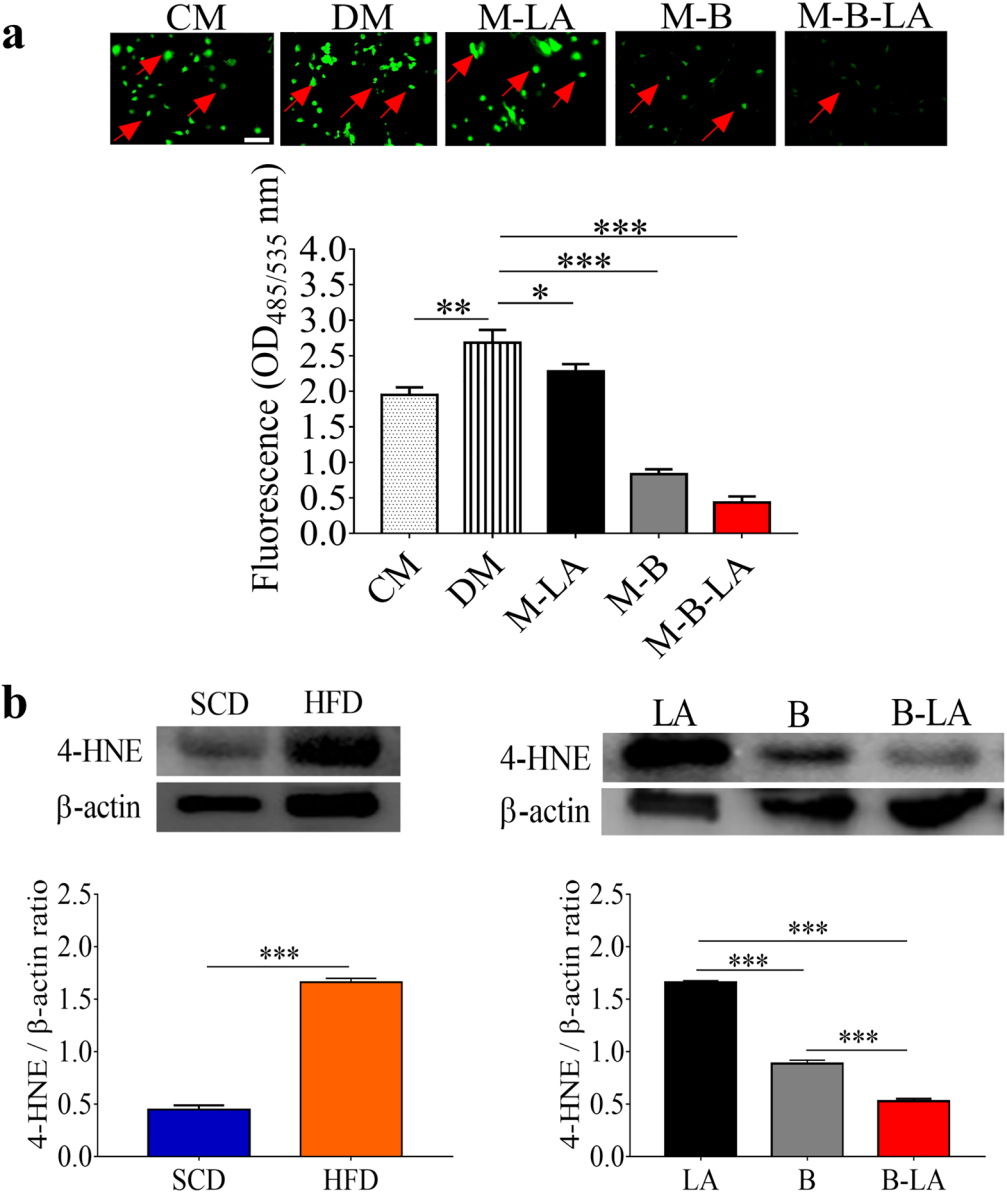
Reduction of the production of ROS and 4-HNE by linoleic acid fermentation of *L. mesenteroides* EH-1. (**a**) Cells loaded with DCFH-DA were treated with media (CM, DM), linoleic acid (M-LA) or supernatants (M-B or M-B-LA) as described in Fig. 2a. Green fluorescence (arrows) derived from DCFH-DA reaction was quantified by measurement with excitation and emission at 485 and 530 nm. (**b**) ICR mice were fed a SCD or HFD at a 3-day interval for 6 weeks. The levels of 4-HNE and β-actin in abdominal fat mass by western blot analysis were quantified by Image J software. The 4-HNE production in abdominal fats in mice administered with linoleic acid alone (LA), *L. mesenteroides* EH-1 bacteria alone (B), bacteria plus linoleic acid (B-LA) was examined. The ratio intensities of 4-HNE to β-actin were quantified by Image J software. Full-length western blot images were presented in Fig. S4. Data are the mean ± SD of experiments performed in triplicate. *p < 0.05. **p < 0.01. ***p < 0.001 (two-tailed *t*-test by GraphPad Prism 5). Bars = 100 µm.

Adipocyte differentiation involves a robust increase in ROS production, leading to oxidative stress^36^. To explore if linoleic acid fermentation of *L. mesenteroides* EH-1 can attenuate ROS induced by adipocyte differentiation, we added the supernatant from the culture of *L. mesenteroides* EH-1 plus linoleic acid onto the differentiating 3T3-L1 preadipocytes. ROS production during 3T3-L1 differentiation was detected by DCFH-DH. ROS was significantly elevated when the culture media of 3T3-L1 preadipocytes were changed to differentiation media. ROS in differentiating cells was greatly suppressed by addition of supernatant of the culture of *L. mesenteroides* EH-1 plus linoleic acid, although suppression of ROS production was also detected by adding the supernatant of the culture of linoleic acid or *L. mesenteroides* EH-1 alone (Fig. 3a).

To evaluate the ability of *L. mesenteroides* EH-1 to attenuate ROS production in vivo, we examined the formation of 4-HNE, a secondary product of oxidative stress^37^, in abdominal fat pads of ICR mice fed with *SCD or* HFD. The level of 4-HNE detected by western blotting in HFD-fed mice was significantly higher than that in SCD-fed mice (Fig. 3b). The level of 4-HNE remained high in mice fed a HFD supplemented with linoleic acid (Fig. 3b). By contrast, the high level of 4-HNE in abdominal fat of HFD-fed mice was markedly reduced when mice were co-administered *L. mesenteroides* EH-1 bacteria alone or bacteria plus linoleic acid by oral gavage (Fig. 3b). Results from high-performance liquid chromatography (HPLC) analysis (Fig. S3) showed that butyrate of greater than 1 mmol/l was detected in cecum of HFD-fed mice administered with *L. mesenteroides* EH-1 alone or plus linoleic acid (Fig. S3). The result suggested butyrate produced by *L. mesenteroides *EH-1 may down-regulate the formation of oxidative stress in differentiating adipocytes and abdominal fat depots.

### Cyclophilin A mediated electricity production of *L. mesenteroides* EH-1

**Figure 4:**
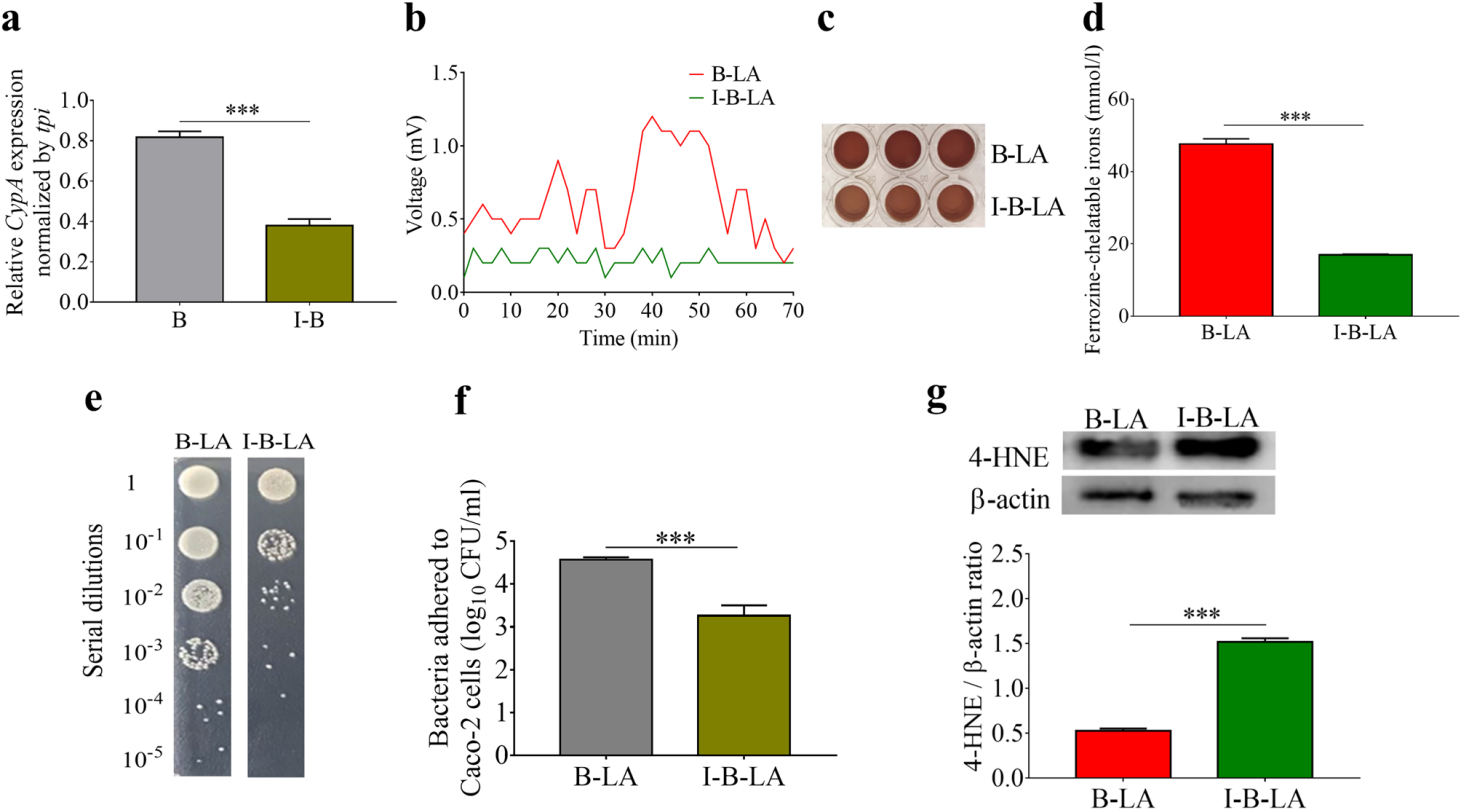
Requirement of *L. mesenteroides* EH-1 cyclophilin A for bacterial electricity and adhesion, as well as reduction of 4-HNE. (**a**) mRNA expression of cyclophilin A (CypA) in *L. mesenteroides* EH-1 pretreated with (I-B) or without TMN355 (B) was detected by real-time PCR. (**b**) Chronoamperometry results were obtained from an electrochemical chamber with *L. mesenteroides* EH-1 pretreated with (I-B-LA) or without (B-LA) TMN355 in the presence of linoleic acid. (**c**) Ferrozine and ferric ammonium citrate were added into media with *L. mesenteroides* EH-1 pretreated with or without TMN355 in the presence of linoleic acid. The dark brown complexes of ferrozine-chelatable irons were photographed. (**d**) The OD_562_ values of ferrozine-chelatable irons (mmol/l) were quantified. (**e**) Adhesion of *L. mesenteroides* EH-1 pretreated with or without TMN355 in the presence of linoleic acid on Caco-2 cells. Bacterial CFUs were counted by plating serial dilutions (1∶10^0^ to 1∶10^5^) of Caco-2 cells with adherent bacteria on a TSB agar plate and (**f**) the number (log_10_ CFU/ml) of adherent bacteria was shown. (**g**) The 4-HNE production in abdominal fats in mice administered *L. mesenteroides* EH-1 plus linoleic acid (B-LA) or TMN355-pretreated bacteria plus linoleic acid (I-B-LA) was examined. The levels of 4-HNE and β-actin in abdominal fat mass were detected by western blot analysis. The ratio intensities of 4-HNE to β-actin were quantified by Image J software. Full-length western blot images were presented in Fig. S5. Data shown represent the mean ± SD of experiments performed in triplicate. ***p < 0.001 (two-tailed *t*-test by GraphPad Prism 5).

FMN-based extracellular electron transfer (EET) is a process of electricity production in Gram-positive bacteria which express peptide pheromone-encoding lipoprotein A on the bacterial membrane. This binds two flavin molecules, enabling electrons to exit the membrane to reach the bacteria’s exterior^38–40^. Although several intracellular molecules or membrane proteins in bacteria function as electron donors or acceptors, respectively, the mediators that transport the electrons from donors to acceptors are not well characterized. Cyclophilin A can sequester cytochrome C, an electron carrier protein^41^. Furthermore, it can bind to peroxiredoxin proteins to support its peroxidase activity as an immediate electron donor^25^. When *L. mesenteroides* EH-1 was pretreated for 24 h with TMN355, a potent cyclophilin A inhibitor, gene expression of cyclophilin A was significantly reduced by about 50% (Fig. 4a). TMN355 itself did not affect the growth of *L. mesenteroides* EH-1 (Fig. S2). To examine whether reduction of cyclophilin A expression influences bacterial electron production, *L. mesenteroides* EH-1 bacteria pretreated with TMN355 were added into media supplemented with 2% linoleic acid in an in vitro chamber with cathode and anode electrodes. Pretreatment of *L. mesenteroides* EH-1 with TMN355 led to a marked attenuation of voltage production relative to bacteria without TMN355 pretreatment (Fig. 4b). This result indicated that cyclophilin A mediated the electricity production of *L. mesenteroides* EH-1. The high concentration of ferrozine-chelatable iron was considerably reduced when the reaction solution contained TMN355-pretreated *L. mesenteroides* EH-1 plus linoleic acid. This result illustrated that *L. mesenteroides* EH-1 plus linoleic acid promoted the reduction of Fe^3+^ to chelated Fe^2+^. Previous studies have shown that electron donors were able to convert Fe^3+^ to Fe^2+^^42,43^. Thus, in this ferrozine assay, electrons produced by *L. mesenteroides* EH-1 plus linoleic acid may use Fe^3+^ as an acceptor to regulate redox cycling of iron.

### Cyclophilin A was essential for bacterial adhesion and reduction of the formation of 4-HNE and abdominal fat depots

A human epithelial *cell* line *Caco-2*, a widely used model of the intestinal epithelial barrier, was ultilized to access whether the electron produced by *L. mesenteroides* EH-1 influenced the bacterial adhesion. Pretreatment of *L. mesenteroides* EH-1 with TMN355 resulted in a significant decrease in the number of bacteria adhered to Caco-2 cell (Fig. 4e,f). We next examined whether inhibition of cyclophilin A altered the ability of *L. mesenteroides* EH-1 to mitigate ROS production in vivo. As shown in Fig. 4g, the level of 4-HNE in abdominal fat of HFD-fed mice administered with TMN355-pretreated *L. mesenteroides* EH-1 plus linoleic acid was noticeably higher than mice administered with *L. mesenteroides* EH-1 plus linoleic acid. Since inhibition of cyclophilin A by TMN355 diminished the electron production, the cyclophilin A-mediated electron production may play a function role in the regulation of bacterial attachment to gut epithelia barrier. In Fig. S3, we have demonstrated that a high amount (> 1.5 mmol/l) of butyrate was produced in cecum of mice administered with *L. mesenteroides* EH-1 plus linoleic acid. Thus, electron mediated by cyclophilin A may facilitate the bacterial adhesion to sustain the high amounts of butyrate for reduction of 4-HNE formation in abdominal fat.

**Figure 5:**
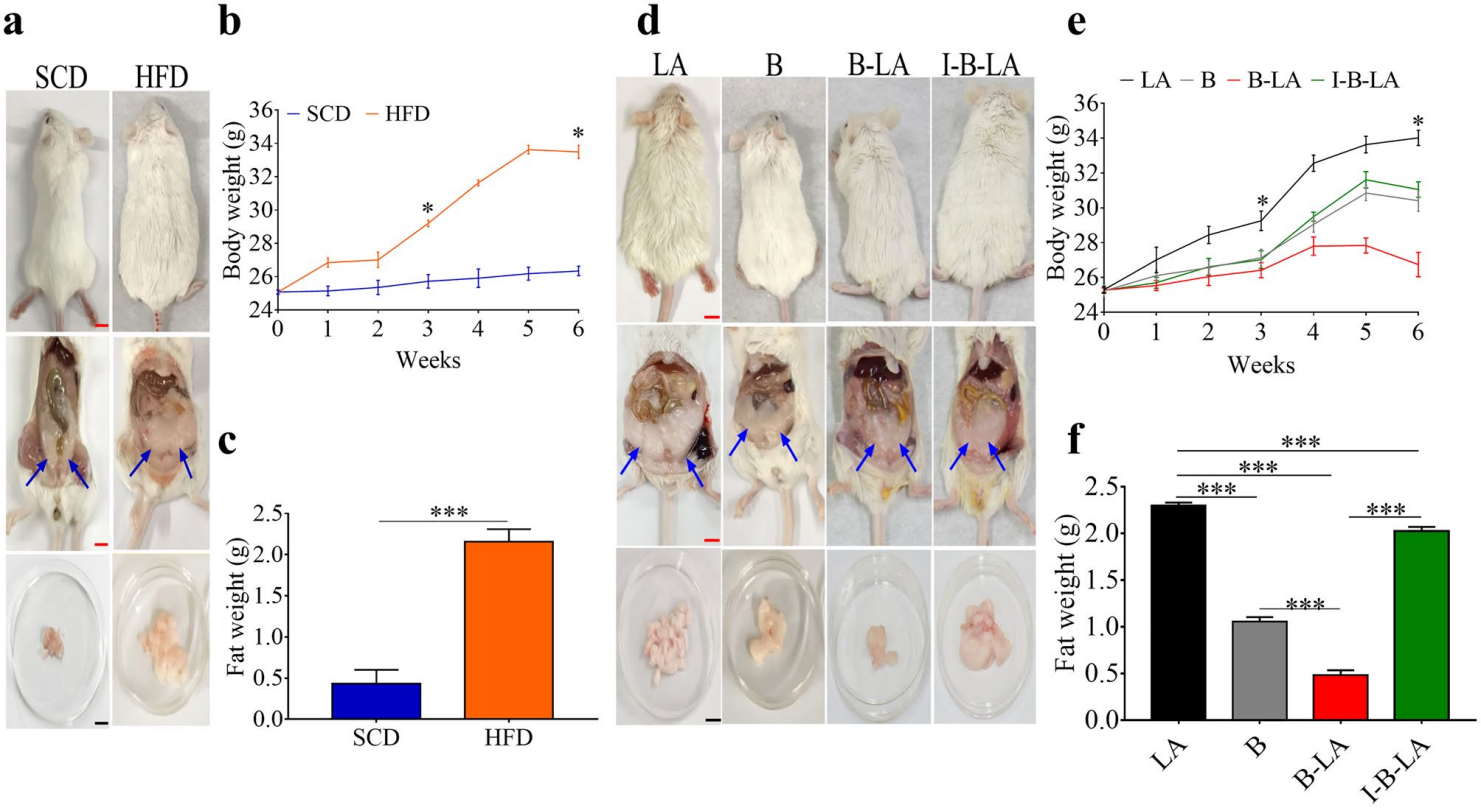
Essential role of electricity produced by bacteria plus linoleic acid in reduction of abdominal fat mass. (**a**) The abdominal fat (arrows) in whole body or isolated abdominal fat masses in mice fed with SCD or HFD was shown. (**b**) Change in body weight was recorded and (**c**) abdominal fat masses were dissected and weighed. HFD-fed ICR mice were administered linoleic acid (LA) alone, *L. mesenteroides* EH-1 bacteria alone (B), bacteria plus linoleic acid (B-LA) or TMN355-pretreated bacteria plus linoleic acid (I-B-LA) for 6 weeks. (**d**) Representative mice with abdominal fat in whole body or isolated abdominal fat masses were shown. (**e**) Change in body weight was recorded and (**f**) abdominal fat masses were dissected and weighted. Scale bars (Black) = 10 mm. Scale bars (Red) = 5 mm. Data shown represent the mean ± SD of experiment in triplicate using five mice per group. *p < 0.05. ***p < 0.001. (two-tailed *t*-tests by GraphPad Prism 5).

We next investigated the consequence of cyclophilin A-mediated electricity on the accumulation of abdominal fat mass in HFD-fed mice. Compared to mice fed with SCD, mice fed with HFD exhibited markedly increased abdominal fat mass (Fig. 5a,c) and body weight (Fig. 5b). Obesity with high body weight was observed in HFD-fed mice receiving orally-administered linoleic acid alone (Fig. 5d,e). Administration of *L. mesenteroides* EH-1 alone, however, caused a reduction of abdominal fat mass (Fig. 5d,f) and body weight (Fig. 5e). This reduction was significantly enhanced when mice were administered *L. mesenteroides* EH-1 plus linoleic acid, resulting in the body weight and fat accumulation similar to that of mice fed with SCD. The reduction of body weight and abdominal fat mass was markedly reduced when mice were treated with TMN355-pretreated *L. mesenteroides* EH-1 plus linoleic acid (Fig. 5d–f). Taken together, results from Figs. 4 and 5 indicated that cyclophilin A-dependent electron generation by *L. mesenteroides* EH-1 regulated lipid accumulation during adipogenesis.

## Discussion

While the electricity produced by *L. mesenteroides* EH-1 is readily detectable in the presence of 2% linoleic acid (Fig. 1a), a low but detectable voltage change was also observed in TSB media containing *L. mesenteroides* EH-1 without addition of linoleic acid. One possible explanation for this detectable electricity is the presence of dextrose in TSB which serves as a potential elecrogenic carbon source. Similarly, although linoleic acid augmented the suppressive effect of *L. mesenteroides* EH-1 on differentiation-induced increase of ROS (Fig. 3a) and lipids in vitro (Fig. 2a) and HFD-induced 4-HNE (Fig. 3b) and abdominal fat masses in vivo (Fig. 5), *L. mesenteroides* EH-1 alone without linoleic acid still can induce some of the same suppressive effects but to a lesser extent. In the absence of linoleic acid, *L. mesenteroides* EH-1 may generate electricity by using other carbon sources for fermentation *L. mesenteroides* EH-1, such as carbohydrates in culture media or the mouse gut. When mice fed with HFD, carbohydrates in HFD can be converted to monosaccharides of glucose, fructose and galactose^44,45^ which will be carbon sources for *L. mesenteroides* EH-1 fermentation to produce electricity and SCFAs.

**Figure 6:**
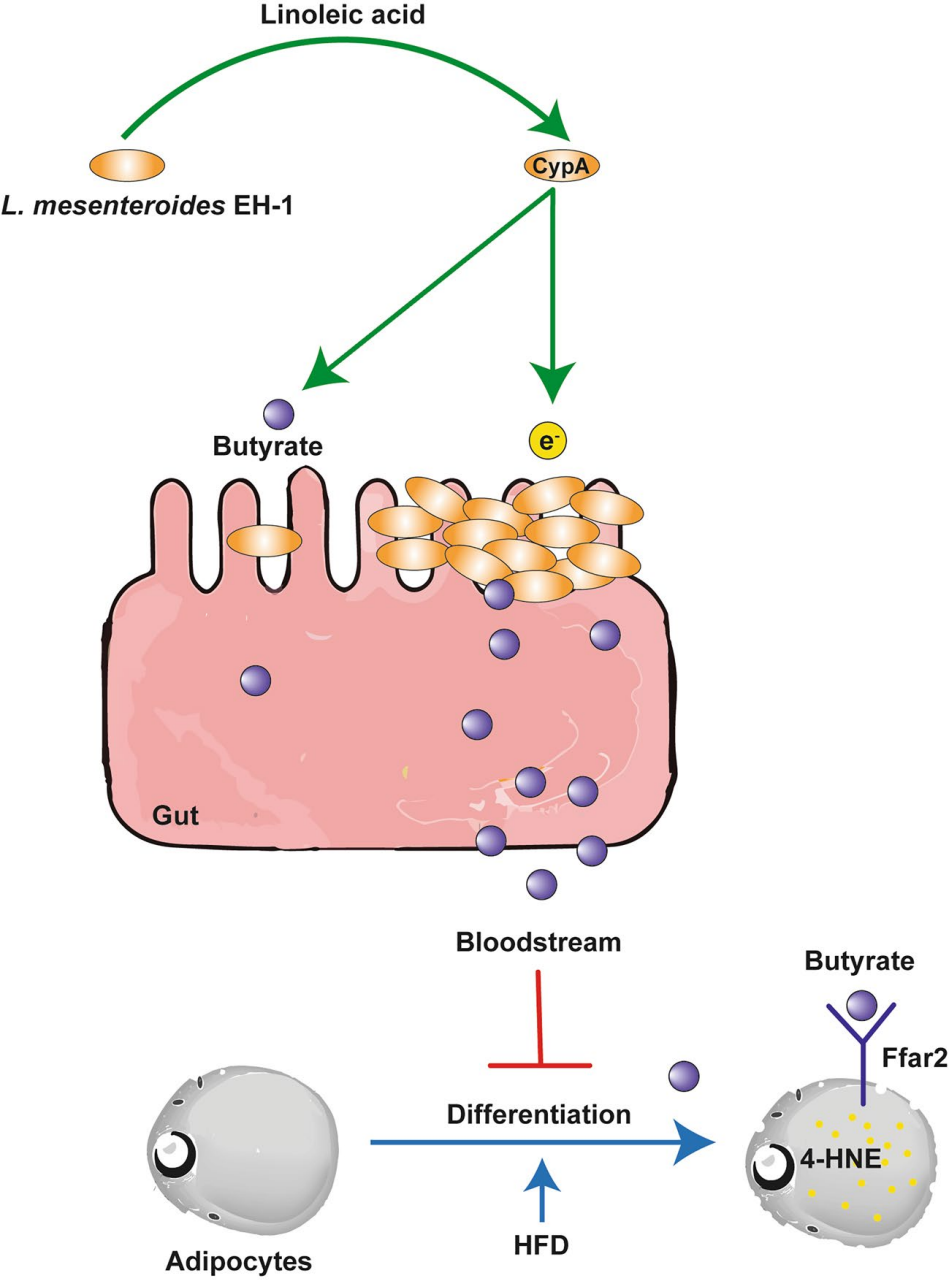
An outline of the actions of *L. mesenteroides* EH-1 on reduction of HFD-induced accumulation of abdominal fat mass. The *L. mesenteroides* EH-1 metabolized linoleic acid to yield electrons (e^−^) via an intracellular cyclophilin A (CypA)-dependent pathway. Electrons enhance bacterial colonization and sustain high amounts of butyrate in the gut. The butyrate may circule through the bloodstream, bind to the Ffar2 on the surface of adipocytes, limit the formation of 4-HNE during adipocyte differentiation, and lower the HFD-induced accumulation of abodominal fat mass. The Adobe Illustrator 2020 (Adobe, San Jose, CA, USA) was used to make this outline.

Gram-positive strains of *Lactobacillus*, *Propionibacterium*, and *Bifidobacterium* bacteria metabolize linoleic acid to vaccenic acid, 10-hydroxy-18:1, and conjugated linoleic acid as a final product which has been found to improve human health^30^. Our results (Fig. 5d–f) demonstrated that feeding mice with linoleic acid alone did not prevent the formation of 4-HNE and abdominal fat masses, suggesting that linoleic acid and its metabolites generated by mouse cells have no effects on adipogenesis. Bacterial fermentation products such as acetate, butyrate, and ethanol can be electron donors^33–35^. It has been reported that acetate and butyrate can attenuate lipopolysaccharide (LPS)-induced lipid peroxidation and ROS^46,47^. Our data demonstrated that *L. mesenteroides* EH-1 used linoleic acid as a carbon source to undergo fermentation (Fig. 1e,f) and produced SCFAs such as acetate and butyrate (Fig. S3). In our previous study, butyrate generated from glucose fermentation by *L. mesenteroides* EH-1 maintained glucose level and enhanced insulin sensitivity^28^. In this study, we have screened the supernatant following linoleic acid fermentation of *L. mesenteroides* (Fig. 1d,e) to quantify their butyrate producing capacity of 0.05 mmol/l by GC–MS analysis (Fig. 1f). However, a higher concentration of butyrate in mice gut was detected as a sharp specific peak in the HPLC chromatogram and was determined to be at a concentration of > 1.5 mmol/l in the linoleic acid fermented media by comparison to a butyrate standard curve (Fig. S3). Blockade of cyclophilin A in *L. mesenteroides* EH-1 by TMN355 limited the electricity production and bacterial adhesion to epithelial cell line Caco*-*2 (Fig. 4). We envision that bacteria produced electron to enhance their colonization on gut epithelial barrier which can lead to the maximum yield of butyrate in the gut. Butyrate may reach adipocytes in abdominal fats via the bloodstream to regulate the accumulation of 4-HNE and fat mass during adipogenesis (Fig. 6).

Cyclophilins are expressed in many tissues and cellular compartments where they act as chaperones to assist protein folding and interaction^48,49^. It has been acknowledged that diverse organisms increase the expression of cyclophilin genes as a defense against oxidative stress^50^. Moreover, cyclophilins can stimulate their antioxidant activity by binding and donating electrons to antioxidant enzymes^25^. Our data demonstrate for the first time that TMN355 down-regulated the expression of cyclophilin A and blocked electricity production (Fig. 4a–d), highlighting the essential role of cyclophilin A in the EET system of *L. mesenteroides* EH-1. Addition of 0.5 mmol/l FMN to the culture of *L. mesenteroides* EH-1 plus linoleic acid significantly enhanced bacterial electricity production (Fig. S1), suggesting *L. mesenteroides* EH-1, as other Gram-positive bacteria, utilize the FMN-based EET system^51^ to yield electricity. Future work will investigate the engagement among cyclophilin A, FMN and other components in the EET system of *L. mesenteroides* EH-1.

Electrons generated by bacterial fermentation are involved in a range of physiological functions^19^. For example, electrons can enhance NAD(P)H and flavoprotein expression, collapse the rate of ROS production, and modulate cell metabolism^52^. Moreover, the role of bioelectricity in the intestinal epithelium has been determined to attract various cells^53^. Interestingly, the extent of the ROS response to enforced electrons may depend on spin-mixing of orbital electron spins with opposite adjacent electron, resulting in a decrease of electrochemical potential^54^. ROS has previously been found to stimulate lipid accumulation during adipocyte differentiation from preadipocytes^55^. It has been reported that the Gram-positive bacteria in mouse gut can mediate EET to produce electricity^19^. Although we cannot exclude the possibility that electrons produced in the gut can travel in the bloodstream to abdominal adipocytes to control adipogenesis, our data demonstrated that butyrate was produced in cecum of mice administered with *L. mesenteroides* EH-1 plus linoleic acid. Butyrate may reach the abdominal adipocytes via bloodstream and eliminate accumulated ROS in differentiated adipocytes. Metabolites such as glutathione (GSH) and SCFAs produced by gut bacteria have been largely recognized to modulate oxidizing conditions toward adipogenesis in adipose tissues^42,56,57^. Our results revealed that electrogenic *L. mesenteroides* EH-1 is a probiotic candidate for suppression of ROS-associated accumulation of abdominal fat mass.

Elevated lipid levels and oxidative stress are the primary pathological processes underlying obesity-related disease. The regulation of cyclophilin A-mediated electricity production in *L. mesenteroides* EH-1 helps alleviate ROS in abdominal adipocytes, successfully ameliorating HFD-induced abdominal fat deposition. Thus, the suppressive effect of *L. mesenteroides* EH-1 on the accumulation of abdominal fat masses can be achieved by eradicating ROS through a novel mechanism associated with butyrate in fermentation production and cyclophilin A-mediated electron production. Although electrogenic bacteria in the gut have been identified^58^ and can be used to predict lymphocyte recruitment^53^, we demonstrate here for the first time that *L. mesenteroides* EH-1 benefits human health by reduction of HFD-induced accumulation of abdominal fat mass.

## Supplementary Information

Supplementary Information.
