# Supplementary material for: Leuconostoc mesenteroides mediates an electrogenic pathway to attenuate the accumulation of abdominal fat mass induced by high fat diet
Source: Sci Rep. 2020 Dec 14;10:21916. doi: 10.1038/s41598-020-78835-9 (PMC7736347; doi:10.1038/s41598-020-78835-9)
Supplement: Supplementary file 1 — Supplementary Information. [file 41598_2020_78835_MOESM1_ESM.docx]

**Supplementary Information**

***Leuconostoc mesenteroides* mediates an electrogenic pathway to attenuate the accumulation of abdominal fat mass induced by high fat diet**

**Minh Tan Pham^1†^, John Jackson Yang^2†^, Arun Balasubramaniam^1^, Adelia Riezka Rahim^1^, Prakoso Adi^1^, Thi Tra My Do^1^, Deron Raymond Herr^3,^ Chun-Ming Huang^1,✉^**

^1^Department of Biomedical Sciences and Engineering, National Central University, Taoyuan, Taiwan

^2^Department of Life Sciences, National Central University, Taoyuan, Taiwan

^3^Department of Pharmacology, National University of Singapore, Singapore, Singapore

^†^ These authors contributed equally to this work

^✉^Correspondence: Professor Chun-Ming Huang. Department of Biomedical Sciences and Engineering, National Central University, Taoyuan 32001, Taiwan. Email: chunming@ncu.edu.tw; Tel:+886-3-422-7151 x 36101; Fax:+886-3-425-3427

Supplementary materials and methods

**Effect of TMN355 on the growth of *L. mesenteroides* EH-1**

*L. mesenteroides* EH-1 (10^7^ CFU/ml) was incubated with or without 2 µM TMN355 in PBS for 24 h. After incubation, bacteria were serially diluted 1:10^0^-1:10^5^ in a 96 well plate. The serially diluted bacteria (10 μl) were dropped on the surface of the TSB agar plate for 9 h. The number of bacteria was determined by counting CFUs.

**Butyrate quantification by HPLC**

Mouse cecum was collected from oral gavage of ICR mice with 50 µl 2% linoleic acid*, L. mesenteroides* EH-1 bacteria (10^7^ CFU) or bacteria plus linoleic acid every day for 3 days. After centrifugation of cecum at 5,000 rpm for 10 min, the supernatants were filtered through a 0.22 μm microfiltration membrane to remove bacteria and all insoluble debris. 100 μl of concentrated HCl was added into filtrates, followed by a vortex mixing step of 15 s. The samples were extracted for 20 min by 5 ml diethyl ether. After centrifugation, the supernatant was relocated to a pyrex extraction tube before adding 500 μl of a 1 mol/l solution of NaOH. The aqueous phase with 100 μl HCl was transferred to an auto-sampler vial. The analysis of butyrate was conducted by an Agilent 1200 series HPLC system with a ZORBAX Eclipse XDB-C18 column (4.6 × 250 mm, 5 μm). The mobile phase contained 20 mM NaH_2_PO_4_ solution (pH 2.2) and acetonitrile. The detector wavelength was 210 nm. The concentrations of butyrate were calculated according to calibration curves of a butyrate analytical standard.

**Supplementary figures**

**
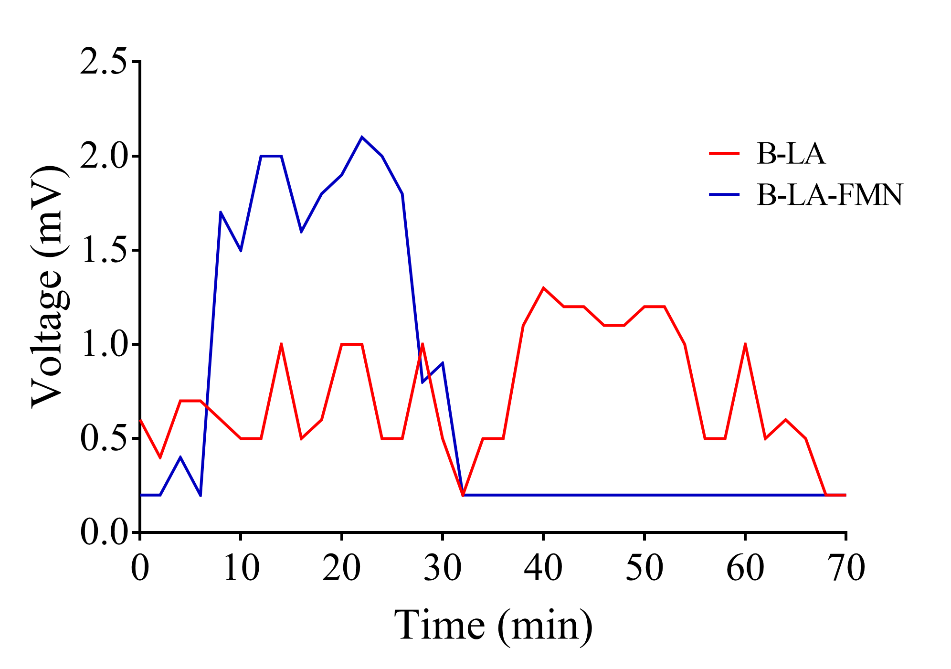
**

**Fig. S1** Electricity enhancement by FMN. Electricity measured by voltage changes (mV) in an *in vitro* chamber was recorded for 70 min in media containing *L. mesenteroides* EH-1 bacteria plus linoleic acid in the absence (B-LA) or presence (B-LA-FMN) of 0.5 mmol/l FMN. The changes in voltage (mV) against time (min) were recorded by Lutron software.

**Fig. S2**

**
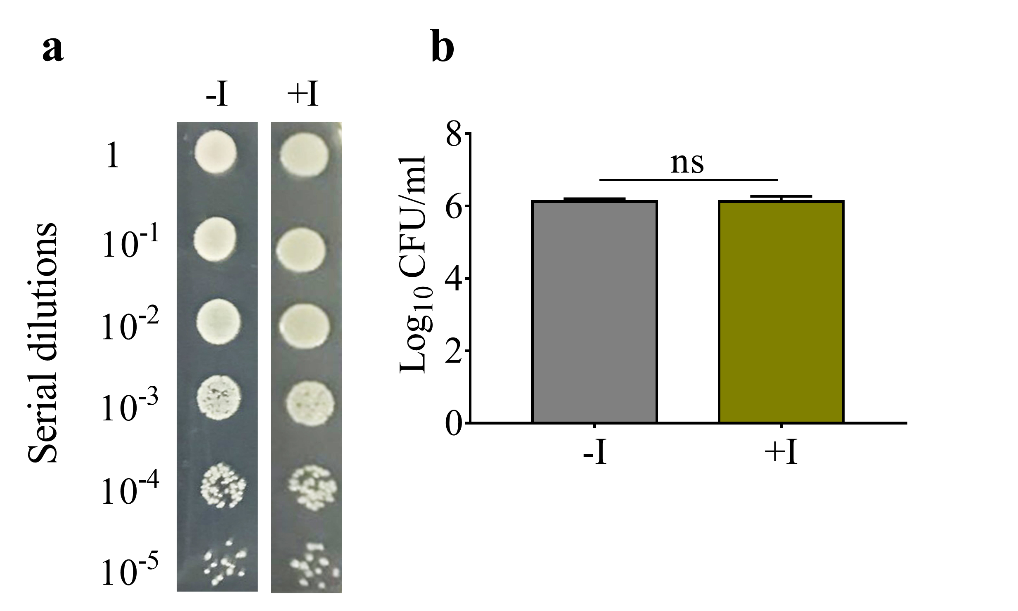
**

**Fig. S2** No effect of TMN355 itself on the growth of *L. Mesenteroides* EH-1*. L. mesenteroides* EH-1 (10^7^ CFU/ml) incubated with (+I) or without (-I) 2 µM TMN355 for 24 h. (**a**) Bacterial CFUs were counted by plating serial dilutions (1∶10^0^ to 1∶10^5^) of the bacterial culture on a TSB agar plate. (**b**) The number (log_10_ CFU/ml) of *L. mesenteroides* EH-1 was shown. Data represent the mean ± SD from results obtained from three independent experiments. ns = non-significant (two-tailed *t*-tests by GraphPad Prism 5).

**Fig. S3**

**
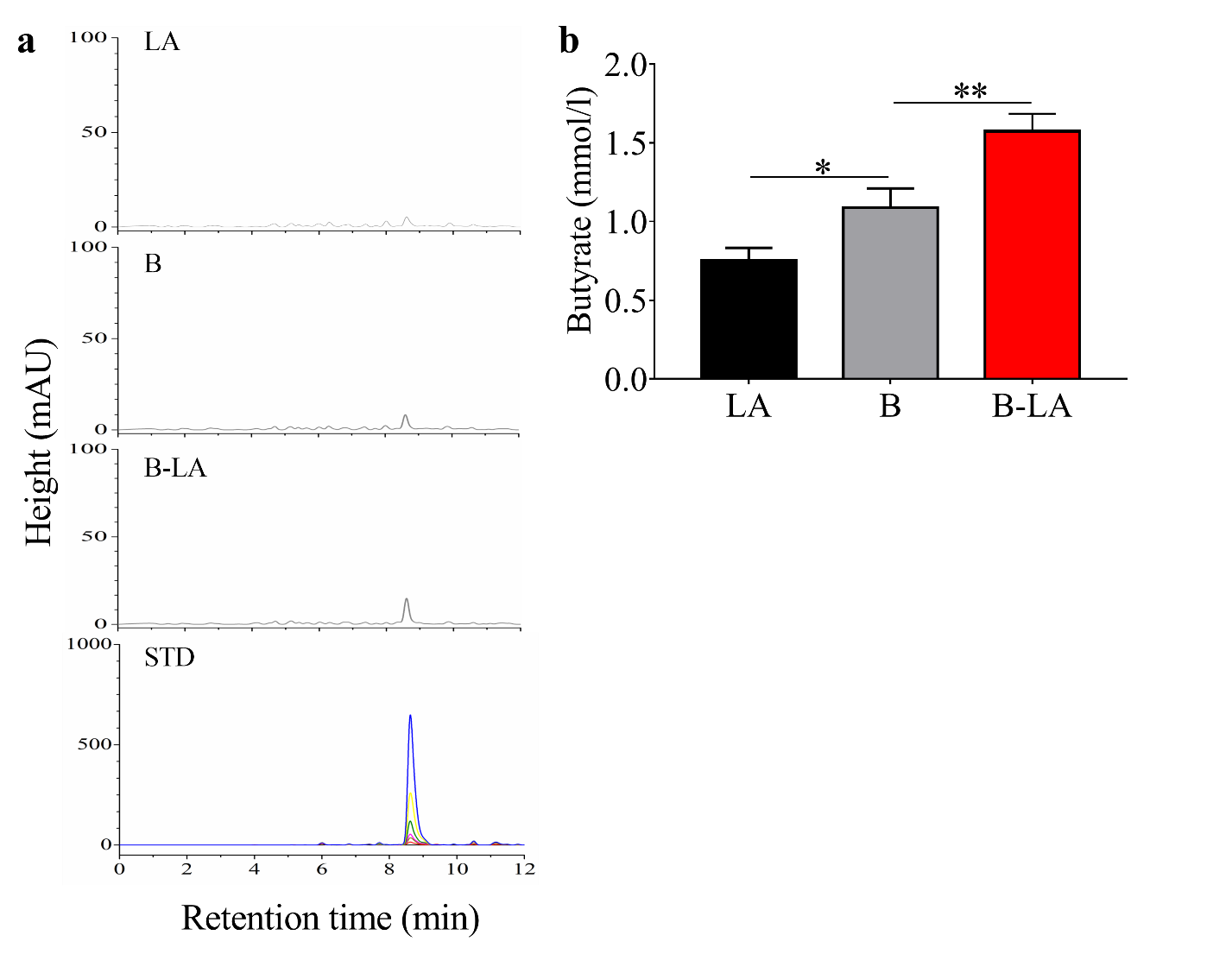
**

**Fig. S3** Butyrate production of *L. mesenteroides* EH-1 in cecum. (**a**) ICR mice were administered with linoleic acid alone (LA)*, L. mesenteroides* EH-1 alone (B), or *L. mesenteroides* EH-1 plus linoleic acid (B-LA) via oral gavage every day for 3 days. The HPLC chromatograms of butyrate in mouse cecum were displayed. (**b**) The concentration (mmol/l) of butyrate was quantified based on the heights [milli-absorbance unit (mAU)] of standard peaks with concentrations of butyric acid from 0-20 mmol/l. Data shown represent the mean ± SD of experiment performed in triplicate. * *p* < 0.05. ** *p* < 0.01 (two-tailed *t*-test by GraphPad Prism 5).

**Fig. S4**


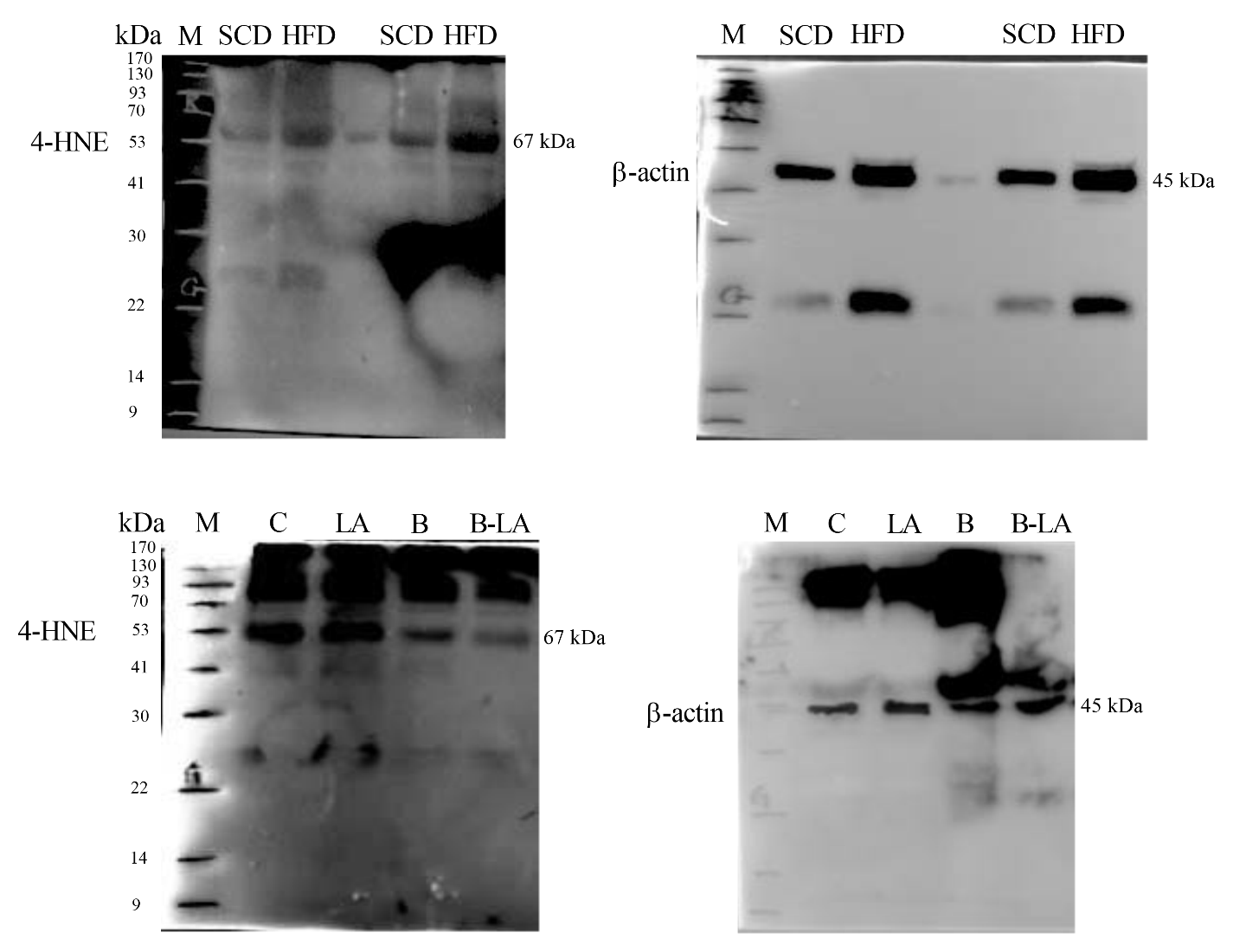


**Fig. S4** The full-length western blot images of Fig. 3b. The upper panels from left to right: Protein markers (M); the protein levels of 4-HNE and β-actin in the abdominal fat mass of mice fed with SCD or HFD. The lower panels from left to right: Protein markers (M); the protein levels of 4-HNE and β-actin in the abdominal fat mass of mice fed with HFD (C) and linoleic acid (LA), *L. mesenteroides* EH-1 bacteria (B) or bacteria plus linoleic acid (B-LA).

**Fig. S5**


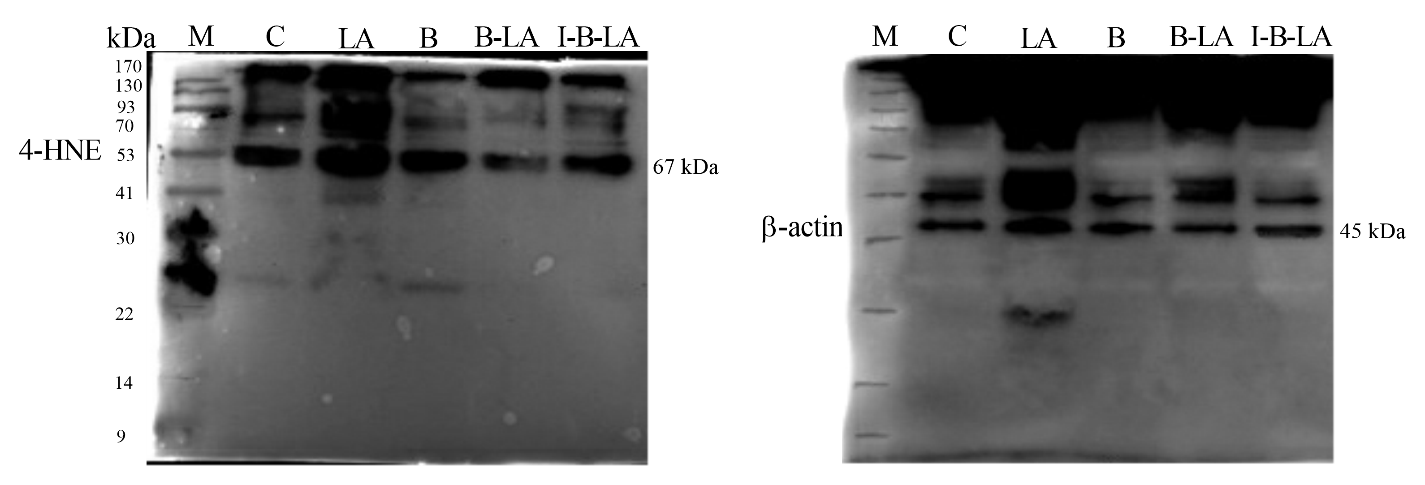


**Fig. S5** The full-length western blot images of Fig. 4g. The lanes from left to right: Protein markers (M); the protein levels of 4-HNE and β-actin in the abdominal fat mass of mice fed with HFD (C) and linoleic acid (LA), *L. mesenteroides* EH-1 bacteria (B), bacteria plus linoleic acid (B-LA) or TMN355-pretreated bacteria plus linoleic acid (I-B-LA).
